# The Impact of Vaccination Status on Anthropometric Indices of Growth Among Children: A Cross-Sectional Study

**DOI:** 10.7759/cureus.64123

**Published:** 2024-07-09

**Authors:** Meha Siddiqui, Naila Bajwa, Khunsa Junaid, Muhammad Awais, Ayesha Amin, Isma Haleem, Sikander H Rasool, Saira Afzal

**Affiliations:** 1 Community Medicine, King Edward Medical University, Lahore, PAK; 2 Community Medicine, Services Institute of Medical Sciences, Lahore, PAK; 3 Community Medicine, Institute of Public Health, Lahore, PAK; 4 Community Medicine, Shaikh Khalifa Bin Zayed Al-Nahyan Medical and Dental College, Lahore, PAK; 5 Public Health and Preventive Medicine, King Edward Medical University, Lahore, PAK

**Keywords:** children, vaccination status, underweight, wasting, stunting

## Abstract

Background

Childhood immunization programs in underdeveloped nations can improve children’s growth and nutritional status and prevent growth delays while protecting against infectious diseases and meeting growth norms. This study aimed to assess the impact of vaccination status on the anthropometric indices of children aged 12-18 months at an Expanded Programme on Immunization vaccination center and compare the anthropometric indices of growth in children with complete and incomplete vaccination statuses.

Methodology

This study was conducted at the rural health center in Kala Shah Kaku, Pakistan, from November 2023 to December 2023. Children aged 12-18 months were enrolled and their vaccination status was recorded. Height and weight were measured using World Health Organization growth charts. The data were analyzed using descriptive statistics and chi-square and Fisher’s exact tests. A p-value <0.05 was considered statistically significant.

Results

The mean age of the 110 children who visited the vaccination site for this study was 16.36 months ± 2.415. There were 28 (25.5%) stunted children. In the study, 17.6 (16%) participants were underweight, and 15.95 (14.5%) were wasted. Of the children, 79% had received all recommended vaccinations. A statistically significant (p < 0.05) association was found between vaccination and nutritional status.

Conclusions

This study emphasizes the significance of vaccination in promoting child health and nutrition, reducing stunting risk, and ensuring equitable access to vaccination services and comprehensive healthcare interventions. This can help mitigate the malnutrition burden and promote optimal growth, contributing to global health and development goals.

## Introduction

Poor child growth is a public health problem in many developing countries, especially in most of the Asian and African regions. In children under five, stunting indicates poor linear growth and chronic undernutrition, whereas wasting indicates acute malnutrition, and underweight is a composite index of both stunting and wasting. Stunted, wasted, and underweight children are defined as the percentage of children 0-59 months old whose height for age, weight for age, and weight for height is below minus two standard deviations from the median of the World Health Organization (WHO) child growth standards. Reduced cognitive development and performance, low intelligence quotients, delayed psychomotor development, lower school attendance, the emergence of chronic diseases such as diabetes mellitus and hypertension, and, ultimately, a loss in the economic and social development of the nation are all consequences of restricted childhood growth. The growth of a child is a sensitive and readily measurable indicator of the health status of children under five years of age. In practice, growth delay in children under five years of age often remains undiagnosed because some children are mostly neither weighed nor measured for height in routine medical visits, and some measures are either not recorded or poorly recorded. Thus, consistent and accurate anthropometric assessment of growing children helps in early identification and timely interventions for emerging health issues [1]. The United Nations Children’s Fund (UNICEF) has highlighted that infectious diseases contribute to poor anthropometric outcomes. This is because it causes reduced dietary intake (for example, loss of appetite, decreased feeding by parents as an effort to end diarrhea), increased nutrient loss (for example, malabsorption and vomiting), and raised nutrient requirements (due to an increase in metabolism as in fever), leading to retarded growth in a child. The most cost-effective health intervention, i.e., childhood immunization, reduces the incidence and duration of infectious diseases, leading to improved child growth [2]. All vaccinations administered during the first year of life are included in basic immunization, which has served as the benchmark for assessing immunization programs. According to the WHO, a child is completely immunized if he/she has received a single dose of bacillus Calmette-Guerin (BCG) vaccine at birth; three doses of oral polio; pentavalent (diphtheria-tetanus-pertussis-hepatitis B (Hep); *Haemophilus influenza* type B (Hib)) at 6, 10, and 14 weeks; and a single dose of measles before 12 months of age [3].

Childhood vaccination coverage has risen globally during the last decades. The coverage of the third dose of the DPT (DPT3) vaccine worldwide is 81.6%, almost double the levels estimated in 1980, which were 39.9%. The worldwide coverage of the first dose of measles (MCV1)-containing vaccine has been raised from 38.5% in 1980 to 83.6% in 2019, while that of a third dose of polio vaccine (pol3) has been raised from 42.6% in 1980 to 79.8% in 2019 [4]. According to estimates from Pakistan, the Expanded Program on Immunization (EPI) coverage varied in different parts of the country, ranging from 65% for combined diphtheria, pertussis, and tetanus (DPT3) and polio to 80% for BCG, with 65% coverage of measles in between [5]. The increase in immunization coverage worldwide has led to the improved growth of children.

According to UNICEF estimates, the prevalence of stunting has declined from 33% to 22% between 2000 and 2020, and the number of children affected has declined from 203.6 million to 149.2 million. Globally, 45 million children under five years of age were estimated to be wasted (low weight for height) in 2021 [6]. Childhood immunization coverage is far below the recommended levels in low- and middle-income countries. The burden of children with poor anthropometric indices of growth is significant in developing countries. A study done in the Mangaung area of South Africa showed that 7.7% of children under five years of age are underweight [7]. In Pakistan, 33% of children under five years of age are underweight, 43.7% are affected by stunting, and around 15.1% by wasting [8]. Immunization is the most essential health intervention against vaccine-preventable diseases. Hence, unvaccinated children or children with incomplete vaccination status are affected by recurrent infectious diseases that hamper their average growth and development and are at risk of dying in their first five years of life [9].

Observational studies across the world have shown an association between vaccination status and the prevalence of underweight, wasting, and stunting [2]. One study showed that underweight (adjusted odds ratio (aOR) = 1.21, 95% confidence interval (CI) = 1.11-1.31), wasting (aOR = 1.18, 95% CI = 1.05-1.33), and stunting (aOR = 1.07, 95% CI = 1.00-1.14) were associated with poor vaccination status [9]. As vaccination prevents infectious diseases, it may also encourage child growth and development. Comprehending the effect of immunization status on children’s growth is essential for developing public health initiatives in our resource-constrained setting, as no such study has been done previously. As our country's immunization schedule ends at 18 months, concentrating on children between the ages of 12 and 18 months facilitates a thorough assessment of the effects of the full vaccination regimen. Therefore, the study aimed to assess the impact of vaccination status on the anthropometric indices of children aged 12-18 months at an EPI vaccination center and compare the anthropometry indices of growth in children with complete and incomplete vaccination statuses. This age group offers a useful and accessible population to assess the impact of vaccination on the anthropometric growth indices of children attending EPI vaccination centers and identify possible areas for intervention and better child health outcomes.

## Materials and methods

Study design

A cross-sectional study was conducted in a rural health center, Kala Shah Kaku, a community setting with primary healthcare.

Sample size calculation

The sample size was calculated using the formula for single population proportion, i.e., n = (Z2pq)/d2, where n is the sample size, z is 1.96, p is 0.627, q is (1-p) = 0.373, and d is the margin of error of 8% [10]. Hence, a sample size of 110 children was taken into consideration.

Sampling method

A consecutive non-probability sampling technique was used to enroll the study participants.

Inclusion and exclusion criteria

Both male and female children aged 12 to 18 months attending the EPI vaccination center at the rural health center with their mothers were included. Children with chronic illnesses or congenital disabilities (such as Down syndrome, a cleft palate, or a musculoskeletal defect) were excluded based on history and general physical examination. Informed consent was obtained from the mothers of children aged 12 to 18 months attending the EPI vaccination center at the rural health center.

Data collection

Mothers with children fulfilling the inclusion criteria were interviewed face-to-face, and a proforma consisting of biodata, anthropometry, and the vaccination status of the children was filled out. The individuals who took interviews and conducted assessments were thoroughly trained before data collection. The completed questionnaires were later cross-checked to ensure the accuracy and honesty of the responses. Biodata included the name of the child, age, gender, father’s occupation, father’s income, and mother’s educational status. The height/length and weight of children were measured using a standardized procedure. A portable digital weighing machine was used to measure the weight (in kg) of children with minimum clothes and the weight was rounded up to the nearest 100 g. Height/length was measured using a non-stretchable measuring tape in a lying position with shoes removed. Using the WHO child growth standard, the height/length and weight measurements were converted into z-scores of the length-for-age, weight-for-age, and weight-for-length, taking the age and sex of a child into consideration. A child with length-for-age, weight-for-age, and weight-for-length below two standard deviations from the median of the WHO standard growth were considered stunted, underweight, and wasted, respectively. Complete essential childhood vaccination was achieved when the child received one dose of BCG vaccine, three doses of pentavalent vaccines, three doses of polio vaccine, and one dose of measles vaccine before the age of 12 months and was categorized as “yes,” while those who failed to take the recommended doses of vaccine were categorized as “no.” The information about child vaccination was extracted from childhood immunization cards to avoid recall bias.

Data analysis

The data obtained were entered and analyzed using SPSS version 26 (IBM Corp., Armonk, NY, USA). The mean and standard deviation were calculated for quantitative variables (e.g., age, height, and weight). Frequency and percentages were calculated for qualitative variables (e.g., vaccination status (complete or incomplete), gender, stunting, underweight, and wasting). Pearson’s chi-square test and Fisher’s exact test were used to assess the association of anthropometric indices of growth with the vaccination status of children. A p-value of less than 0.05 was considered significant in the bivariate analysis. Further, stratification of stunting, wasting, and underweight was done for the number of siblings, father’s income, and mother’s educational status to control confounders, and a post-stratification chi-square test was performed.

Ethical considerations

Participants provided verbal informed consent and were informed about the study’s aims, methods, and benefits. The study was approved by the Committee of Research Ethics at King Edward Medical University, Lahore, Pakistan (approval number: 319/RC/KEMU) with minimal potential risks and the right to withdraw without reprisals.

## Results

This study included a total of 110 children attending the EPI vaccination center. The mean age of the study participants was 16.36 ± 2.415 months. The mean weight of the participants was 9.11 ± 1.237 kg, and the mean height was 22.20 ± 4.573 cm. The study involved 110 participants, with 36 (32.7%) males and 74 (66.3%) females. Overall, 28 (25.5%) of children were stunted, 16 (14.5%) were wasted, and 18 (16.6%) were underweight. Moreover, 81 (79%) children were fully vaccinated (Table 1).

**Table 1:** Demographic characteristics of the participants (N = 110).

Variables		Frequency (n)	Percentages (%)
Gender	Male	36	32.7
	Female	74	67.3
Number of siblings	0–2	76	69.1
	3–5	34	30.9
	>5	0	0
Father’s occupation	Laborer	57	51.8
	Government employee	30	27.3
	Private service/Business	23	20.9
Father’s income per month	<50,000 Rs	41	37.3
	50,000–100,000 Rs	42	38.2
	>100,000 Rs	27	24.5
Mother’s educational status	Illiterate	9	8.2
	Primary	9	8.2
	Secondary	24	21.8
	Matric	22	20
	FA	12	10.9
	Bachelors	34	30.9
Stunting	Yes	28	25.5
	No	82	74.5
Wasting	Yes	16	14.5
	No	94	85.5
Underweight	Yes	18	16.4
	No	92	83.6
Normal	Yes	61	55.5
	No	49	44.5
Vaccination status	Complete	87	79.1
	Incomplete	23	20.9

Stunting was found to be more frequent among males, that is, 18 (64.2%) males, while 10 (55.5%) females were stunted. Moreover, stunting was found to be more prevalent in children whose fathers were laborers by occupation, that is, 26 (92.8%). Wasting was found more in females; 13 (81.25%) female children were wasted, while only three (18.75%) male children were wasted (Table 2).

**Table 2:** Anthropometric growth indices in children by their sociodemographic factors (N = 110).

Socioeconomic and demographic factors	Stunting, N (%)		Wasting, N (%)		Underweight, N (%)	
	Yes	No	Yes	No	Yes	No
Gender						
Male	18 (64.2)	18 (21.95)	3 (18.75)	33 (35.10)	5 (27.77)	31 (33.7)
Female	10 (55.5)	64 (78.04)	13 (81.25)	61 (64.8)	13 (72.2)	61 (66.3)
Number of siblings						
0–2	12 (42.8)	64 (78.1)	7 (43.75)	69 (73.4)	2 (11.11)	74 (80.4)
3–5	16 (57.1)	18 (21.9)	9 (56.25)	25 (26.5)	16 (88.88)	18 (19.5)
Father’s occupation						
Laborer	26 (92.8)	31 (37.8)	9 (56.25)	48 (51)	16 (88.88)	41 (44.6)
Government employee	0	30 (36.5)	6 (37.5)	24 (25.5)	0	30 (33.3)
Private service/Business	2 (7.1)	21 (25.6)	1 (6.25)	22 (23.4)	2 (11.11)	21 (22.8)
Father’s income						
<50,000 Rs	19 (67.8)	22 (26.8)	9 (56.25)	32 (37.8)	16 (88.88)	25 (27.1)
50,000–100,000 Rs	7 (25)	35 (42.6)	6 (37.5)	36 (36.5)	0	42 (45.65)
>100,000 Rs	2 (7.1)	25 (30.4)	1 (6.25)	26 (25.6)	2 (11.11)	25 (27.1)
Mother’s educational status						
Illiterate	8 (28.5)	1 (1.2)	1 (6.25)	8 (8.5)	4 (22.22)	5 (5.4)
Primary	4 (14.2)	5 (6.09)	2 (12.5)	7 (7.4)	1 (5.5)	8 (8.6)
Secondary	7 (25)	17 (20.7)	7 (43.75)	17 (18)	6 (33.33)	18 (19.5)
Matric	0	22 (26.82)	5 (31.25)	17 (18)	5 (27.7)	17 (18.4)
FA	7 (25)	5 (6.09)	1 (6.25)	11 (11.7)	2 (11.11)	10 (10.8)
Bachelors	2 (7.1)	32 (39)	0	34 (36.1)	0	34 (36.9)

**Figure 1:**
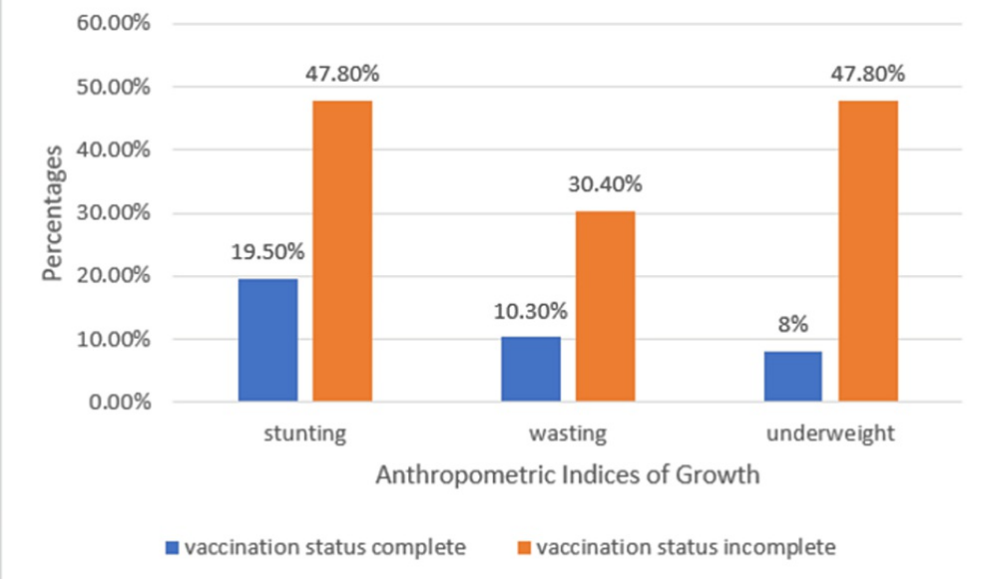
Anthropometric indices of growth in children based on their vaccination status (N = 110).

Incompletely vaccinated children had higher rates of stunting, wasting, and being underweight compared to fully vaccinated children, with 11 (47.8%) being stunted, seven (30.4%) being wasted, and 11 (47.8%) being underweight among incompletely vaccinated children (Figure 1).

The p-value for stunting, wasting, and being underweight, when compared with vaccination status, was found to be statistically significant in children with incomplete vaccination status (p < 0.05) (Table 3).

**Table 3:** The relationship between anthropometric indices of growth and vaccination status (N = 110).

Anthropometric indices of growth	Vaccination status	Yes		No		Chi-square value	P-value
		N	%	N	%		
Stunting (low height-for-age)	Completely vaccinated	17	19.5	70	80.5	7.67	0.006
	Incompletely vaccinated	11	47.8	12	52		
Wasting (low height-for-weight)	Completely vaccinated	9	10.3	78	89.6	5.9	0.015
	Incompletely vaccinated	7	30.4	16	69.5		
Underweight (low weight for height)	Completely vaccinated	7	8	80	91.9	21.03	0.000
	Incompletely vaccinated	11	47.8	12	52		

Post-stratification chi-square analysis revealed statistically significant associations with a p-value less than 0.05 for the following: stunting with the number of siblings (3-5) and mother’s educational status (primary); wasting with female gender and mother’s educational status (secondary); and underweight with the number of siblings (3-5), father’s income (<50,000 Rs), and mother’s educational status (illiterate and secondary education).

## Discussion

The study examines the vaccination status of children aged 12-18 months at the EPI vaccination center, rural health center, Kala Shah Kaku. Overall, 79% of children were completely vaccinated, which is consistent with a previous study showing a drop in the global coverage for the DTP3 vaccine from 86% in 2019 to 81% in 2021 [11]. In our study, 28 (25.5%) children were found to be stunted. This is low compared to another study conducted in India that reported a 44.82% pooled prevalence of stunting [12]. Eastern and middle Africa have the highest prevalence in the UN sub-regions, at 50% and 42%, respectively [13]. These community-based studies included both vaccinated and non-vaccinated preschool children, and these are the countries where vaccination coverage of children under five years of age is low.

The developed countries where awareness of vaccination is higher and nearly all children get recommended vaccines have comparatively lower prevalence rates, as shown in a study from Latin America, where the prevalence of stunting was as low as 1.8% in Chile, 7.1% in Brazil, and 8.2% in Argentina [14]. Overall, 16 (14.5%) children were found to be underweight in our study. Likewise, the reported prevalence of underweight children was 22.7% in Ethiopia [15]. The reported pooled prevalence of underweight (as per WHO standards) children in India was 42.96% [13]. On the other hand, the prevalence of underweight children was very low in developed regions such as Europe. It was found to be 12%, 6.8%, 6.7%, and 11% in Eastern, Northern, Southern, and Western Europe, respectively [16]. The study found that 15.5% of children were wasted, a significant difference from India’s 23.69% and Ethiopia’s 22% prevalence [13,15]. In developed countries, where parents are motivated to get their children vaccinated, the prevalence of stunting is extremely low. In China, only 1.2% of children were wasted, a significant difference from developing countries. Developing countries, such as China, have a higher rate of wasting and stunting [16,17].

The study revealed an association between immunization status and stunting in children aged 12-18 months. Stunting was more common in unvaccinated or partially vaccinated children than in vaccinated children. This aligns with previous research showing a link between nutritional status and immunization status [18-20]. Vaccines are crucial for preventing infectious diseases that can worsen malnourishment and stunted growth. Vaccinated children may experience low appetite, nutrient malabsorption, and stunted growth. Vaccines protect children from diseases such as measles, pneumonia, and diarrhea, reducing the risk of stunting and promoting healthy growth [21]. Moreover, vaccination status could be used as a stand-in for the adoption of preventive health activities and access to healthcare services. Having received all recommended vaccinations, children are more likely to see doctors regularly and have access to essential medical services such as growth tracking, dietary counseling, and supplementation plans. These services are essential for locating and treating the underlying causes of stunting, which include poor food intake, deficiency in certain micronutrients, and viral illnesses [22].

This study explored the association between wasting and vaccination status among children. Wasting, characterized by low weight-for-height, is a significant indicator of acute malnutrition and is linked to increased morbidity and mortality rates in children under five years old [23]. Vaccination status, on the other hand, indicates a person’s defense against infections that can be prevented by vaccination. However, some studies have found no correlation or alternative patterns of association between wasting and incomplete vaccination status [24]. This discrepancy may be due to variations in study populations, settings, and methodologies, as well as factors such as socioeconomic level, dietary habits, and access to healthcare services. Regardless of vaccination status, fully vaccinated children may have better access to healthcare services and preventive treatments, potentially reducing the risk of wasting.

The study used standardized and validated measurement techniques to accurately assess children’s anthropometric indices, ensuring consistency and validity. It recorded vaccination status from vaccination cards to avoid the mother’s recall bias. Potential confounders such as the father’s income, occupation, and mother’s educational status were controlled to minimize their effect on the association. The study fills a gap in existing literature in resource-limited settings. It suggests ways to meet vaccination and nutritional requirements, such as enhancing vaccination outreach efforts, integrating health services, educating parents, monitoring vaccination rates, advocating for policy changes, and conducting research on the lasting impact of vaccination status on child development. These efforts aim to improve health outcomes for children under two by boosting vaccination rates.

However, it is essential to acknowledge certain limitations of our study that may have influenced the observed relationship between anthropometric indices of growth (stunting, wasting, and underweight) and vaccination status. The study’s cross-sectional design limits causal relationships between variables, necessitating longitudinal studies to understand the temporal sequence and mechanisms. The sampling technique used may have led to selection bias, as participants from vaccination centers may not accurately represent the population. Further research in a community setting is needed to produce more valid findings, as individuals visiting a vaccination center may have access to healthcare or be more health-conscious.

## Conclusions

The study reveals that children’s vaccination status and anthropometric growth are significantly linked with children’s attendance at vaccination centers. Lower vaccination rates lead to stunting. Socioeconomic factors such as education, income, and occupation also contribute to poor growth. Improving parents’ literacy, family socioeconomic status, and access to vaccination services can help mitigate malnutrition and promote healthy child growth, ultimately contributing to achieving health and development goals.
